# Regulation of Developmental Cell Death in the Animal Kingdom: A Critical Analysis of Epigenetic versus Genetic Factors

**DOI:** 10.3390/ijms23031154

**Published:** 2022-01-21

**Authors:** Juan A. Montero, Carlos Ignacio Lorda-Diez, Juan M. Hurle

**Affiliations:** Departamento de Anatomía y Biología Celular and IDIVAL, Universidad de Cantabria, 39011 Santander, Spain; carlosignacio.lorda@unican.es

**Keywords:** apoptosis, programmed cell death, necrosis, caspase, lysosome, senescence

## Abstract

The present paper proposes a new level of regulation of programmed cell death (PCD) in developing systems based on epigenetics. We argue against the traditional view of PCD as an altruistic “cell suicide” activated by specific gene-encoded signals with the function of favoring the development of their neighboring progenitors to properly form embryonic organs. In contrast, we propose that signals and local tissue interactions responsible for growth and differentiation of the embryonic tissues generate domains where cells retain an epigenetic profile sensitive to DNA damage that results in its subsequent elimination in a fashion reminiscent of what happens with scaffolding at the end of the construction of a building. Canonical death genes, including Bcl-2 family members, caspases, and lysosomal proteases, would reflect the downstream molecular machinery that executes the dying process rather than being master cell death regulatory signals.

## 1. Historical Background

In addition to the turnover of tissues subjected to permanent renewal, cell death was considered in the past an undesirable event in healthy pluricellular animals. It was largely assumed that cell death in adult animals was a pathological phenomenon associated with degenerative diseases, ischemia, mechanical damage, or toxin exposure. The term “*necrosis*” was coined to name this process, assigning several adjectives to differentiate some processes from others according to the outcome of the damaged tissue (coagulative, liquefactive, caseous, etc.). Lysosomes, as a source of hydrolytic enzymes, are considered central, but often passive, effectors of these degenerative processes (see [1]). However, in the first half of the last century, researchers became aware of the presence of massive degenerative processes associated with the growth and differentiation of embryonic and larval organisms. Remarkably, these processes were closely correlated with morphological (i.e., tail loss during tadpole metamorphosis), structural (i.e., formation of neural circuits in the developing CNS), or functional (i.e., maturation of the immune system) modeling of developing organisms, suggesting that in embryonic systems, cell death reflected developmentally regulated planning (see reviews [2,3]). Consistent with this view, the existence of a dead clock within the prospective dying cells of some embryonic organs was proposed to determine the time when cells should die [4]. However, this dead-clock was not confirmed in other models of programmed cell death where cells can be experimentally diverted from the death program, giving rise to ectopic functional structures [5,6].

## 2. Apoptosis versus Necrosis

The identification of intense cell dying processes in actively growing tumors and during involution of hormone-dependent tissues, together with the morphological similitudes between embryonic dead cells and such tumor dying cells, and the absence of inflammatory response accompanying these degenerative processes were taken as evidence for a specific form of cell death, distinct to *necrosis*, that was termed *apoptosis* [1,7]. Among the morpho-structural features that differentiate apoptosis from necrosis, the following can be highlighted: apoptotic cells appear rounded, they preserve the integrity of the membranes, and in the nucleus the chromatin appears densified with clumps of greater density in their contour. In contrast, necrotic cells appear swollen and in the course of disintegration with a massive rupture of the plasma membrane and vacuolization of the organelles. Initially, apoptosis was thought to be an active genetically programmed degeneration involving the activation of endogenous endonucleases [8], while necrosis, was considered a passive process resulting from circumstances outside the cell.

## 3. Genetic Regulation of Cell Death

The hypothesis of a genetic regulation of developmental cell death became intensely reinforced by the identification of a number of genes in the nematode *Caernohabditis elegans* (*C. elegans*), termed “cell death abnormal genes” (*Ced* genes) whose mutation abolished the physiological dying process occurring in the course of development of this worm [5].

The results obtained from the study of an “oncogene” associated with the human B cell lymphoma, termed *Bcl-2*, gave a great push to the concept of apoptosis as a genetically regulated process (see [9]). It was found that this gene protected cells from death and was highly homologous, and functionally interchangeable with the *C. elegans Ced-9* gene [10,11,12]. *Bcl-2* was the first gene associated with cell death in mammalian cells, but soon a complex regulatory network constituted by a large family of proteins sharing one or various characteristic domains of BCL-2, termed BH domains (BCL-2 homology domains), was discovered.

Another *C. elegans* cell death abnormality gene, Ced-3, which is crucial for the execution of cell death in the worm and is found downstream of Ced-9 in the death cascade [13], was found to be homologous to a family of cysteine-aspartic proteases, called caspases, which orchestrate most of the steps of the apoptotic process in vertebrate organisms. The apoptotic caspases include *initiator caspases*, occupying initial or intermediate steps in the degeneration cascade (caspase 2, 8, 9, and 10 in humans) and *executioner caspases* (caspases 3, 6, and 7) that complete the degeneration process. The functions of executioner caspases include the cleavage of distinct structural and regulatory proteins and the activation of caspase-dependent endonuclease that in turn break the DNA at the internucleosomal spaces [8].

## 4. Intrinsic and Extrinsic Apoptotic Pathways

Detailed studies in a wide variety of models established the occurrence of two pathways of activation of the apoptotic molecular cascade: one originating from extracellular signals belonging to the TNF superfamily (*extrinsic apoptotic pathway*) and the other originating inside the cells, *intrinsic, or mitochondrial, apoptotic pathway* (see Galluzi et al. [14] for a detailed review). The latter being the predominant mechanism responsible for developmental cell death in vertebrates.

The intrinsic pathway is triggered by distinct, but often related, endogenous cell perturbations that include DNA damage, deprivation of growth factors, or increase in reactive oxygen species (ROS). Such perturbations trigger a complex functional interplay between BCL-2 family members that results in the permeabilization of the outer mitochondrial membrane by the BH-multidomain factors, BAX, BAK, and BOK and subsequent cytosolic release of damaging factors, including Cytochrome C, AIF (apoptotic inducing factor), DIABLO (direct IAP-binding protein with low pI), or HtrA2 serine proteases. The activation of BAX and BAK is regulated by a balance between a positive (pro-apoptotic) influence of members of the BCL-2 family containing the BH3-domain only (BIM, BID, PUMA, and NOXA), and a negative (antiapoptotic) influence of members of the family containing the four BH domains (BCL-2, BCL-Xl, MCL1, BCL-W, BFL-1). The cytochrome C delivered from the mitochondria exerts a key role in the next step of the apoptotic cascade generating active caspase 9 via binding to APAF-1 (apoptotic peptidase activating factor 1) and pro-caspase 9 to form the so-called *apoptosome*. Finally, caspase 9 catalyzes the activation of executioner caspases.

The extrinsic pathway is triggered by the binding of TNF family ligands (TNF alpha, FASL, and TRAIL) to transmembrane receptors that contain a characteristic intracellular death domain (FAS, TNFR1, DR4, and DR5). Upon ligand binding the intracellular tail of the receptor forms a multiprotein complex termed DISC (death-inducing signaling complex) that directs the activation of caspase 8. This caspase may proteolytically activate the executioner caspases and/or activate the intrinsic pathway via proteolytic activation of BID.

Considering the complexity of these dying cascades associated with embryonic programmed cell death, apoptosis was often considered an evolutionarily conserved cell self-destruction process (see [15]). However, all the members of the apoptotic cascades also exert functions unrelated to cell death [16,17]. The existence of universal activators of apoptosis and transcription factors functionally specialized to direct cell death have been suggested in *C. elegans* [18] and *Drosophila* [19] but has not been identified in developing vertebrates. In fact, it would be difficult to explain the evolutionary conservation of a gene whose expression results in the disappearance of the organism that possesses it. Consistent with this interpretation, mouse phenotypes following silencing components of the apoptotic molecular cascade reveal a limited impact of apoptosis deficiency in normal development [20,21] (and see below).

## 5. Multiple Varieties of Non-Apoptotic Cell Death

Intensification of research in recent decades has provided a more complex view of cell degeneration processes in both embryonic and adult systems [14]. Rather than *necrosis* and *apoptosis* only, different forms of cell death of distinct biological significance and regulated by specific triggering mechanisms were identified (see [14,22]). In some cases, such as the so-called *pyroptosis* or *necroptosis*, the degenerating cells exhibit intermediate features between necrosis and apoptosis; in other cases, lysosomes play the leading role in the degenerative processes.

The initial proposal of “apoptosis” as a regulated process alternative to necrosis ruled out the role of lysosomes, considering them responsible for passive cell death induced by external damaging agents or implicated in the phagocytic removal of the cell debris generated in the degenerative process. However, the systematic study of different models of developmental cell death allowed for the identification of dying mechanisms in which lysosomes exerted a major function. In fact, prior to the consolidation of the term apoptosis, Schweichel and Merker [23], based on transmission electron microscopic observations, proposed the terms “necrosis type I”, for what was later termed apoptosis, “necrosis type II”, for dying processes characterized by the implication of lysosomes via autophagy, and, finally, “necrosis type III” for what now is called necrosis. With the advance of genetic and molecular technologies to study cell death, lysosomes were confirmed as major effectors of cell death [24,25].

There are, at least, two alternative lysosomal ways for accomplishing cell self-destruction. Destruction may be mediated via permeabilization of the lysosomal membrane followed by release of lysosomal hydrolases into the cytosol (lysoptosis [26,27]) or may destroy the cells via intensification of autophagy, as originally described for type II cell death [23]. However, autophagy, was traditionally considered a survival mechanism that provides energy to cells subjected to metabolic stress [28]. Consistent with this view, in some cases lysosomal activation accompanies, but does not cause, the degenerative process [29], and in other cases, rather than inducing cell death autophagy, protects cells from dying [28].

Remarkably, in recent years, *developmental cell senescence* has been proposed as a new tissue remodeling mechanism [30], but, most likely, it could in fact represent the destructive process formerly assigned to autophagy [31]. As autophagy, senescence was originally considered a defense mechanism against cell injuries, and is characterized by three features: (1) cell cycle arrest via upregulation of tumor suppressor genes; (2) the elaboration of a senescence-associated secretory phenotype (SASP) that promotes the local expansion of senescence; and (3) lysosomal activation detectable via histochemical detection of senescence-associated-β- galactosidase (SAβ-gal) [31] or cathepsin D [32]. All these features take place together in embryonic areas of programmed cell death, and histochemical detection of SAβ-gal at pH 6 overlaps with traditional markers for developmental cell death, such as Nile blue, or neutral red vital staining, which detect lysosomes in the areas of cell death [33,34,35,36]. Furthermore, chemical inhibition of cathepsin D reinforces the inhibition of programmed cell death mediated by caspase inhibitors [37]. Based on these findings, it has been proposed that *developmental cell senescence* represents the involvement of lysosomes in programmed cell death, receiving the name of *destructive cellular senescence* to be distinguished from the canonical form of senescence as a cellular defense mechanism [31].

Of note, lysosomal implication in cell death may act individually or in concert with the apoptotic molecular machinery [38,39,40]. Regardless lysosomal activity occurring via cytosolic release of proteases or by its delivery into the autophagic vacuoles, the required permeabilization of lysosomes appears to be mediated by the multidomain members of the BCL-2 family, BAX and BAK [25]. In addition, cathepsins delivered after permeabilization of lysosomes may cleave and activate initiator or executioner caspases, or promote mitochondrial outer membrane permeabilization [38,41,42], thus activating in a cooperative fashion lysosomal cell death and caspase-dependent cell death.

A singular mechanism that escape from the conventional cell autonomous regulation of dying process results from the ingestion of still alive cells by their neighbors. This dying mechanism has been described in cancer studies with the name of entosis [43]. In this case, it is considered that the prospective dying cell invades a non-phagocytic neighbor cell prior to undergoing degeneration. A relative similar process has also been reported in developmental models with term of “assisted suicide” [44], but different to entosis, the elimination of the dying cells involves a true phagocytic process. The mechanisms underlying these processes are out of the scope of our review.

The discovery of different types of cell deaths included the identification of a large panel of dying executioner factors in addition to the well-known mitochondria-delivered factors and executioner caspases [45], and the multifunctional properties of crucial effectors of apoptotic cell death that are also able to trigger necrosis [45].

## 6. Redundancy of Dying Mechanisms in Developing Systems

Since the establishment of the concept of programmed cell death, this process has been considered not only of major importance in morphogenesis but also a causal mechanism of abnormal development induced by teratogens [46]. However, the scarcity of overt cell death phenotypes following genetic ablation of the different components of the cell death molecular cascades in the mouse questioned the occurrence of a major developmental function for cell death unless the dying mechanism was very redundant [21]. Thus, mice subjected to single gene silencing of distinct caspases [47] or Bcl-2 family members [21] lack major morphological alterations in organs sculpted by physiological cell death. Similar negative findings were found after genetic ablation of lysosomal cathepsins [48,49]. Furthermore, independent chemical inhibition of caspases or lysosomal proteases caused only a low inhibition of interdigital cell death responsible for sculpting the digits from the embryonic autopod [27]. However, mouse genetic studies [50] and observations in the embryonic chick model supported the functional redundancy of dying machinery [51]. It was observed that the partial inhibition of interdigital cell death by local pancaspase inhibitor treatments was potentiated when the treatment was combined with inhibitors of lysosomal proteases [27]. Furthermore, it was also observed that mouse KO for caspase 9, which plays a crucial role in apoptosis, does not have digit phenotypes because under this experimental condition, interdigital cells die via necrosis instead of apoptosis [50].

Combined mutations of proapoptotic members of the Bcl-2 family reinforced the redundant nature of the dying machinery. Double or triple KOs of pro-apoptotic Bcl-2 superfamily members, such as Bax and Bak, exhibit cell death phenotypes, such as syndactyly [52], but the inhibition of cell death and subsequent syndactyly appears to be incomplete, as the penetrance of the phenotype is potentiated by additionally silencing genes associated with autophagy [53].

## 7. Sensitivity of the Prospective Areas of Programmed Cell Death to Cell Damaging Agents

A common observation in former teratological studies was that the undifferentiated tissue regions of the embryo, such as those undergoing physiological cell death, were the most prone to be altered by diverse damaging stimuli [46] (virus embryopathy, ionizing irradiation, etc.). Often, teratogen exerts its effect by inducing new cell death regions [54] or by increasing the extension of the areas of physiological cell death [55]. This association can explain why, frequently, in old teratological studies, normal areas of cell death were mistakenly taken as abnormal [46]. The specific sensitivity of embryonic regions to cell damage was confirmed in studies of chick embryos subjected to sublethal X-irradiation [56]. Thus, in avian embryos, sublethal X-irradiation at stages preceding the formation of free digits causes massive degeneration of the interdigital cells preceding the appearance of the physiological areas of interdigital cell death without altering digit development. Remarkably, as that which occurs physiologically, cell death is preceded by DNA damage detectable by immunohistochemical detection of γH2AX foci that are precocious markers of DNA repair.

## 8. Epigenetic Profile and Embryonic Programmed Cell Death

The above surveyed data and advances in the study of programmed cell death in recent decades support the following contentions: (1) no master transcription factors responsible for triggering cell death in vertebrate embryos have been identified; (2) cells fated to die retain their potential to differentiate and survive until the initiation of the dying process (i.e., there is not a “dead clock”); (3) dying pathways are multiple and functionally redundant; and (4) all components of the dying machinery have functions unrelated to cell death. Considering these facts together, it can be concluded that the initiation of embryonic dying processes rather than being dependent on a dying-specific signal is caused by increasing the sensitivity of the target cells to damage by signals that are not harmful for their neighboring cells destined to survive.

Information accumulated in the last decade points to epigenetic modifications and chromatin remodeling as crucial regulators of embryonic cell behavior by facilitating or hindering the access of transcription factors to their targets and regulating chromatin fragility. This type of regulatory mechanism would explain why a signal may be active for a particular cell but not for close neighbor cells. In a complementary fashion, this would also explain the occurrence of distinct or even antagonistic responses to a particular signal among closely related cells. However, the precise basis for the differential sensitivity to damage should not necessarily be identical in different embryonic contexts. Thus, blastomeres of mammalian embryos at the two-cell stage are protected from apoptosis due to DNA methylation and histone deacetylation, which make the chromatin inaccessible to DNAses activated by caspase 3 [57]. However, in an opposite fashion, increased DNA methylation via upregulation of DNA methyltransferases has an intense proapoptotic influence in motor neurons of postnatal and adult mice [58] and in photoreceptors of mouse models of retinitis pigmentosa [59].

During amphibian anura metamorphosis, thyroid hormone (TH) is a crucial regulatory signal that promotes growth in some tadpole tissues (i.e., limb primordia) and, at the same time, massive degeneration in other tissues [60] (tail, gills, gut, etc.). The effects of TH depend on the regulation of the activity of histone deacetylases by corepressor or coactivator factors that result in chromatin remodeling accompanied by transcriptional activation or inhibition of target genes in stage- and organ-specific manners [61,62].

Further evidence for a role of epigenetics as an initial step of programmed cell death comes from studies of the dying process responsible for the separation of the digits during limb development in tetrapods. The interdigital remodeling process is carried out by massive cell death involving caspases and lysosomal activation in a redundant fashion [31,63]. Consistent with the activation of caspases and lysosomal proteases, interdigital dying cells show morphological features of apoptosis, senescence, necrosis and, autophagy [31]. Of note, preceding the activation of the dying machinery, the interdigital progenitors bear increased genome instability to X-irradiation in comparison with their neighboring digit-forming tissue and show spontaneous DNA damage and repair activity detectable by immunolabeling of phosphorylated histone 2AX at serine 139 (γH2AX) [31]. In the course of interdigit remodeling, γH2AX- foci associate with zones of intense DNA methylation and histone 3 trimethylation at lysines 4, 9, and 27 (H3K4me [3]; H3K9me [3]; and H3K27me [3]) [56,64] suggesting that regions of elevated DNA fragility depend on chromatin architectural cues. Consistent with this interpretation, major epigenetic regulators responsible for DNA methylation, such as UHRF1 (ubiquitin-like containing plant homeodomain and RING finger domain), DNA methyltransferases (*Dnmt1*, *Dnmt* 3a, and *Dnmt* 3b), and different histone deacetylase genes (*Hdac1*, *Hdac2*, *Hdac3*, and *Hdac8*), show regulated expression domains in the interdigital regions [56,65,66]. Experimental analysis designed to unravel the relevance of those expression domains on cell death revealed a dramatic increase in interdigital cell death in vivo following local inhibition of histone deacetylases with trichostatin A [65,67]. In addition, cell death was increased and decreased in primary cultures of limb skeletal progenitors after overexpression or silencing of the *Dnmt3b* gene, respectively [56]. Remarkably, overexpression and silencing of *Dnmt3b* promotes and inhibits the pattern of DNA methylation across the promoter of SOX9, a master gene of chondrogenesis with a major influence on chromatin remodeling that is required for the survival of limb skeletal progenitors [68,69] and mesenchymal stem cells [70].

As mentioned above, a general epigenetic modification rule accounting for cell death in different systems cannot be expected, as distinct modifications may favor cell death in distinct cell populations. Among the epigenetic signatures proposed to be associated with normal or abnormal developmental dying processes are histone modifications [71,72,73,74] and/or promoter methylation of specific genes, including master transcription factors [75,76], genes encoding secreted factors [77], or tumor suppressor and cell death regulatory genes [78,79].

## Data Availability

Not applicable.
